# Coverage with evidence development schemes for medical devices in Europe: characteristics and challenges

**DOI:** 10.1007/s10198-021-01334-9

**Published:** 2021-06-12

**Authors:** Carlo Federici, Vivian Reckers-Droog, Oriana Ciani, Florian Dams, Bogdan Grigore, Zoltán Kaló, Sándor Kovács, Kosta Shatrov, Werner Brouwer, Michael Drummond

**Affiliations:** 1 Centre for Research On Health and Social Care Management, SDA Bocconi School of Management, Bocconi University, Via Roberto Sarfatti 25, 20100 Milan, Italy; 2 School of Engineering, Warwick University, Coventry, UK; 3 Erasmus School of Health Policy and Management, Erasmus University Rotterdam, Rotterdam, The Netherlands; 4 KPM Center for Public Management, University of Bern, Bern, Switzerland; 5 Swiss Institute of Translational and Entrepreneurial Medicine (Sitem-Insel AG), Bern, Switzerland; 6 Evidence Synthesis and Modelling for Health Improvement, Institute of Health Research, College of Medicine and Health, University of Exeter, Exeter, UK; 7 Syreon Research Institute, Budapest, Hungary; 8 Erasmus School of Economics, Erasmus University Rotterdam, Rotterdam, The Netherlands; 9 Centre for Health Economics, University of York, York, UK

**Keywords:** Coverage with evidence development, Medical devices, European HTA policies, Value of information, Adoption and reimbursement of medical devices, I18

## Abstract

**Objectives:**

Medical devices are potentially good candidates for coverage with evidence development (CED) schemes, as clinical data at market entry are often sparse and (cost-)effectiveness depends on real-world use. The objective of this research was to explore the diffusion of CED schemes for devices in Europe, and the factors that favour or hamper their utilization.

**Methods:**

We conducted structured interviews with 25 decision-makers from 22 European countries to explore the characteristics of existing CED programmes for devices, and how decision makers perceived 13 pre-identified challenges associated with initiating and operating CED schemes for devices. We also collected data on individual schemes that were either initiated or still ongoing in the last 5 years.

**Results:**

We identified seven countries with CED programmes for devices and 78 ongoing schemes. The characteristics of CED programmes varied across countries, including eligibility criteria, roles and responsibilities of stakeholders, funding arrangements, and type of decisions being contemplated at the outset of each scheme. We observed a high variability in how decision makers perceived CED-related challenges possibly reflecting country-specific arrangements and different experiences with CED. One general finding across all countries was that relatively little attention was paid to the evaluation of schemes, both during and at their completion.

**Conclusions:**

CED programmes for devices with different characteristics exist in Europe. Decision-makers’ perceptions differ on the challenges associated with these schemes. More exchange of knowledge and experience will help decision makers anticipate the likely challenges in CED schemes for devices, and to learn from good practices existing elsewhere.

**Supplementary Information:**

The online version contains supplementary material available at 10.1007/s10198-021-01334-9.

## Introduction

At the time of the publication of the ISPOR ‘Good Practices for Performance-Based Risk-Sharing Arrangements (PBRSAs) Task Force’ report [1], it was acknowledged that there were two types of arrangements to aid the market entry of new technologies; finance-based and performance-based agreements. Briefly, in finance-based arrangements, agreements between payers and manufacturers are purely financial and may involve for example price–volume agreements, price discounts or budget caps. In PBRSAs one of the key elements is that the price, or reimbursement of a technology is linked to its performance which is assessed through a purposeful, prospective data collection.. Indeed, some of the earliest examples of PBRSAs concern coverage with evidence development (CED) schemes that were initiated by the Centres for Medicare & Medicaid Services in the US and the Ontario Ministry of Health in Canada for medical procedures and devices [2, 3]. In CED schemes, data are collected with the objective of reducing uncertainty concerning the clinical or cost-effectiveness of a health technology and to assist in future decisions about its reimbursement, coverage, or recommendations for its use. Typically, these schemes are centrally coordinated and require substantial data collection.

Since the publication of the Task Force’s report, two trends can be observed. Firstly, there has been a growth in the popularity of finance-based agreements, or simple price reductions, as compared with performance-based schemes. In a recent review of managed entry agreements in Europe, Dabbous et al. [4] note that ‘despite the interests in CED schemes, European countries have moved towards finance-based agreements due to the complexities and burdens associated with PBRSAs’. The lack of appetite for complex agreements among policy-makers was also noted by Karlsberg Schaffer et al. [5], who concluded that ‘there is a mismatch between the enthusiasm in the academic literature for developing new approaches and the scepticism of payers that they can work, or are necessary for the foreseeable future’. Secondly, there has been a growth in the application of financed-based and performance-based agreements to drugs rather than to other types of technologies, which could be a response to the growing number of transformational, but highly expensive new drugs entering the market [6]. Many recent reviews of PBRSAs discuss issues that apply to health technologies in general but draw almost exclusively on drugs for their examples [4, 7–9].

In principle, medical devices are good candidates for PBRSAs, particularly for CED schemes, since there are often considerable uncertainties concerning their (cost-)effectiveness. This is mainly because the data requirements to obtain market access are often less stringent than those for drugs, and therefore devices are generally adopted in clinical practice with relatively little clinical or economic evidence [10, 11].. In contrast to pharmaceuticals where the market authorization and supervision is centrally managed by the European Medicine Agency (EMA) (Regulation (EC) No 726/2004), the conformity assessment procedures for medical devices of risk class II or higher in Europe are decentralized and operated by public or private notified bodies (NBs) which are designated by the EU member states. Evidence requirements for market authorization are regulated by the medical device regulation (MDR), which also defines when a clinical investigation of the new device is required or when conformity assessment can be based on the equivalence principle with a previously marketed device. However, notwithstanding the requirements for clinical investigations, a controlled clinical trial, which demonstrates the relative effectiveness compared to alternative treatments, is generally not mandatory for MDs. Besides the differences in the regulatory approaches compared with pharmaceuticals, certain sources of uncertainty around a medical device are relatively less easy to explore by means of pre-market studies. Many devices are part of complex interventions, consisting of multiple behavioural, technological, and organizational components, and therefore their actual (cost-)effectiveness profile usually depends on a series of context-specific factors that are difficult to assess before their adoption in the real-world. For example, device performance in regular clinical practice often depends not only on the device itself, but also on the skills of the user [12, 13]. In addition, while finance-based agreements are also possible, the cost of adopting a new device depends not only on its price, but also the cost of any new procedures or other organizational changes that might be required for its use. Therefore, a price reduction for the device itself may have less of an impact on overall costs. Moreover, finance-based agreements do not resolve potential issues about uncertainty in the effectiveness of the device, which both payers and patients may feel is important.

The pace of innovation in medical devices is considerable, with many new products entering the market every year. For example, in 2017, the number of patents in the field of medical technologies filed with the European Patent Office (EPO) was more than double compared to the number concerning pharmaceuticals (13,000 versus 6300), and the total expenditure on medical technologies in Europe was roughly estimated as €115 billion [14]. Given the relevance of the market and the above-mentioned challenges with evidence generation at market launch, any policy tool such as CED, which foresees a controlled introduction of a technology while collecting further post-market evidence, is highly relevant in the context of medical devices. However, despite the possible advantages of CED schemes for aiding coverage decisions regarding new devices, little is known about the extent to which these schemes are used in Europe and the detailed perceptions of decision-makers regarding their utilization [15–17]. Therefore, the objective of this research was to contribute to filling this gap by exploring the characteristics and diffusion of CED schemes for devices in Europe, and the challenges that decision-makers face during the different phases of a scheme [1, 14]. Our aim was to assist those considering the implementation of CED schemes for medical devices and to increase the understanding of both how schemes are currently being applied in Europe and how the challenges associated with them are being addressed.

## Methods

This study is part of the EU Horizon 2020 COMED project that has been reviewed and approved by the Bocconi University Ethics Committee (protocol number: 0068538, approved on May 8, 2018).

The research was conducted in three consecutive steps: (1) development of a structured interview guide (2) interviews with decision-makers from a sample of European countries, (3) synthesis and qualitative content analysis of the interview data, the data made available by the decision-makers during or following the interview, and data on scheme characteristics previously obtained [17]. The steps are described in more detail below.

### Development of the interview guide

We developed a structured interview guide (Online Resource 1) that consisted of three sections. Section A included general questions on whether CED programmes underpinning the individual schemes existed in the decision-maker’s country and for which type of technology they were used. Section B included questions on 13 challenges for CED schemes for devices (Table 1). This list was derived from a recent systematic review that identified 20 challenges for CED schemes for devices [17]. To reduce the participants’ burden, we reduced the original list of 20 challenges to 13, by grouping different aspects of the same general challenge. The final list of challenges was discussed and agreed among all authors to ensure that all relevant aspects originally identified were covered in the interview guide (see the Online resource 2 for more details).

**Table 1:** phases of CED schemes

Assessing the desirability of a scheme	
This initial phase relates to the way candidate technologies for CED schemes are identified and selected. It also concerns the criteria used to assess whether a scheme is a good policy option, compared with other available options such as, for example, fully adopting the technology despite the residual uncertainties; refusing to adopt the technology until better evidence becomes available; or negotiating/mandating a lower price for the technology.	
Designing the scheme	
This phase is about deciding on the specific features of the scheme design. These include, for example, the categories of patients who will have access to the technology during the scheme (e.g., Only in Research or Only With Research schemes), and the characteristics of the data collection plan, such as the study design (e.g., registry-based studies *versus* randomized controlled studies), the duration of the data collection, and the types of outcomes to be measured.	
Implementing the scheme	
Reflecting the previous design phase, this phase is about the different ways schemes are operated and how roles and responsibilities are distributed among the stakeholders involved (e.g., the national/regional HTA agencies, the manufacturers, or the providers collecting the data). Relevant aspects are, for example, who will initially design the study protocol, who will coordinate and/or perform the data collection, monitoring and analysis, and who will fund the provision of care and the extra costs of collecting the new evidence.	
Evaluating the scheme	
This phase relates to the types of decisions/policy updates that are made at the end of the scheme once the data collection is concluded and the new evidence has been assessed along with other evidence that has become available. It also concerns the way data collection is monitored during the scheme and the definition of any stopping rule or intermediate assessment of the evidence being collected	

We asked the decision-makers to assess how they perceived the 13 challenges to apply to CED schemes for devices on a six-point Likert scale (ranging from 0 “not a challenge” to 5 “a major challenge”). Where CED schemes for devices existed, we also asked respondents how the challenges were met in their country, and the interview proceeded to Section C. Otherwise, the interview ended here. Section C included questions on the detailed characteristics of individual CED schemes for devices that had been either initiated or still ongoing in the past five years. These questions concerned a description of the device under evaluation, its clinical application, the objective of the scheme, key sources of uncertainty, funding of the scheme, its design, the decision rule, and outcome (if re-assessment was done), and any public source of information on the scheme.

### Interviews with decision-makers

A first draft of the interview guide was circulated for comments among the COMED project partners. Subsequently the final draft of the interview guide was pilot tested during interviews with one Italian policy maker and two academic experts with extensive experience of CED in Canada and the USA, two countries with a substantial number of schemes.

The interviews were conducted face-to-face or by telephone between June and December 2019. Decision-makers from decision bodies at the central (or in two cases regional) level were identified from the professional networks of the members of the COMED project team or the websites of relevant decision bodies in the following European countries: Austria, Belgium, Bulgaria, Croatia, Czech Republic, Denmark, England, Finland, France, Germany, Greece, Hungary, Iceland, Ireland, Italy, the Netherlands, Norway, Poland, Portugal, Romania, Scotland, Slovakia, Slovenia, Spain, Sweden, and Switzerland. Other countries from the EU/EEA were excluded because it was not possible to identify a relevant decision-making body for the technology assessment of medical devices. We invited decision-makers to participate in the study by sending them an email with information on the COMED project and the objective of our study. When we were unable to identify a decision-maker from the networks or websites, we sent the information and invitation to the relevant decision bodies. In three cases where no relevant decision-maker could be identified (i.e. Bulgaria, Czech Republic, and Sweden), we invited academic researchers with relevant expertise to participate. None of these countries had however any CED programme for devices in place. We interviewed more than one decision maker from a given country in cases where schemes were operated in more than one jurisdiction (i.e., Italy), where more than one decision body was involved in operating schemes (i.e., France), or where more than one decision-maker, from different parts of the relevant organization, agreed to participate (i.e., England). We excluded Croatia, Iceland, Romania, and Slovenia from our sample after repeated attempts to schedule an interview by December 2019 were unsuccessful. Information on the individual CED schemes provided by decision-makers during or following the interview was supplemented with information on individual schemes previously obtained [17], compiled in tabular form, and sent to the participants for a validity check.

### Data analysis

The transcripts were subjected to qualitative content analysis using deductive coding to meet the objective of this research. The results of each interview were reported in a table by one author (CF) and assessed by two authors (CF and VRD) who independently extracted the relevant information. Agreement on the data to be reported was then reached through discussion and further analysis of the original transcripts. The data obtained from Sections A and C of the interview guide, together with the data obtained prior to and following the interviews were used to identify and classify the characteristics of the existing CED programmes for devices according to the four phases of CED schemes: 1) assessing the desirability of the scheme; 2) designing the scheme; 3) implementing the scheme, and 4) evaluating it (1). These phases are described in more detail in Table 2. The information collected was then synthesised in a narrative review.

**Table 2:** Challenges with CED schemes for medical devices^a^

	Challenge
1	Deciding which medical devices are candidates for CED schemes
2	Obtaining stakeholder agreement on the scheme
3	Securing funding for the scheme
4	Determining the appropriate study design for data collection
5	Determining the relevant outcome measure(s) on which data are collected
6	Dealing with data collection and monitoring
7	Dealing with data analysis
8	Ex-ante definition of decision rule, based on possible outcomes of the scheme
9	Reaching an agreement on price, reimbursement or use of the device at the end of the scheme
10	Withdrawing a device from the market when evidence indicates the device is not (cost-) effective
11	Obtaining agreements about the duration of the scheme and the stopping rule
12	Adapting the scheme to account for product modifications or a learning curve
13	Dealing with the market entry of similar devices

*CED* coverage with evidence development

^a^Derived from Reckers-Droog et al. [17]

The data obtained from Section B of the interview guide were used to obtain insight into the participants’ perceptions of the 13 challenges and into the factors that influenced their score for a particular challenge. The quantitative data obtained from Section B were used to calculate the mean (SD) and median (IQR) Likert scores for the 13 challenges (excluding the challenges that were marked as ‘not applicable’ by the participants). Then, we calculated these statistics separately for participants from countries with and from countries without a CED programme for medical devices. Because of the small sample sizes, we did not examine the differences in scores by performing statistical tests, but all factors which were perceived as having a positive or negative influence on each challenge were synthetized in tabular form.

## Results

We interviewed 25 participants from 23 jurisdictions. Respondents were from national or regional health authorities (*n* = 15); national health insurance bodies (*n* = 2); hospitals (*n* = 3); and universities (*n* = 3) (see Online Resource 3 for details). Eighteen participants had high-level managerial roles related to the HTA of medical devices or services, or were responsible for the CED programme in their jurisdiction; four participants were technical advisers directly involved in the assessment of medical devices, and three were academics with an expertise in conditional reimbursement schemes. In seven out of the 23 jurisdictions (30.4%), CED programmes existed that included (or were specific to) schemes for medical devices (i.e., Belgium, England, France, Germany, the Netherlands, Spain, and Switzerland). In France, two different programmes were identified: Post Registration Studies (PRS) for devices submitting for registration into the positive list of reimbursable products and services (LPPR list); and Forfait innovation (FI) for highly innovative technologies early in their development phase. Of the remaining jurisdictions, 5 (21.7%) operated CED programmes for drugs only (i.e., Bulgaria, Hungary, Portugal, Scotland, and Slovakia), and 11 (47.8%) did not operate any CED programmes (i.e., Austria, Czech Republic, Denmark, Finland, Greece, Ireland, Italy-Emilia Romagna Region, Italy-national level, Norway, Poland and Sweden), although some of these may have other types of PBRSAs such as performance linked reimbursement schemes (e.g., payment by results schemes). In addition, single ‘one-off’ experiences with schemes for specific devices were reported by participants from Emilia Romagna Region in Italy and Ireland, in the absence of formal programmes for CED schemes for devices.

**Fig. 1:**
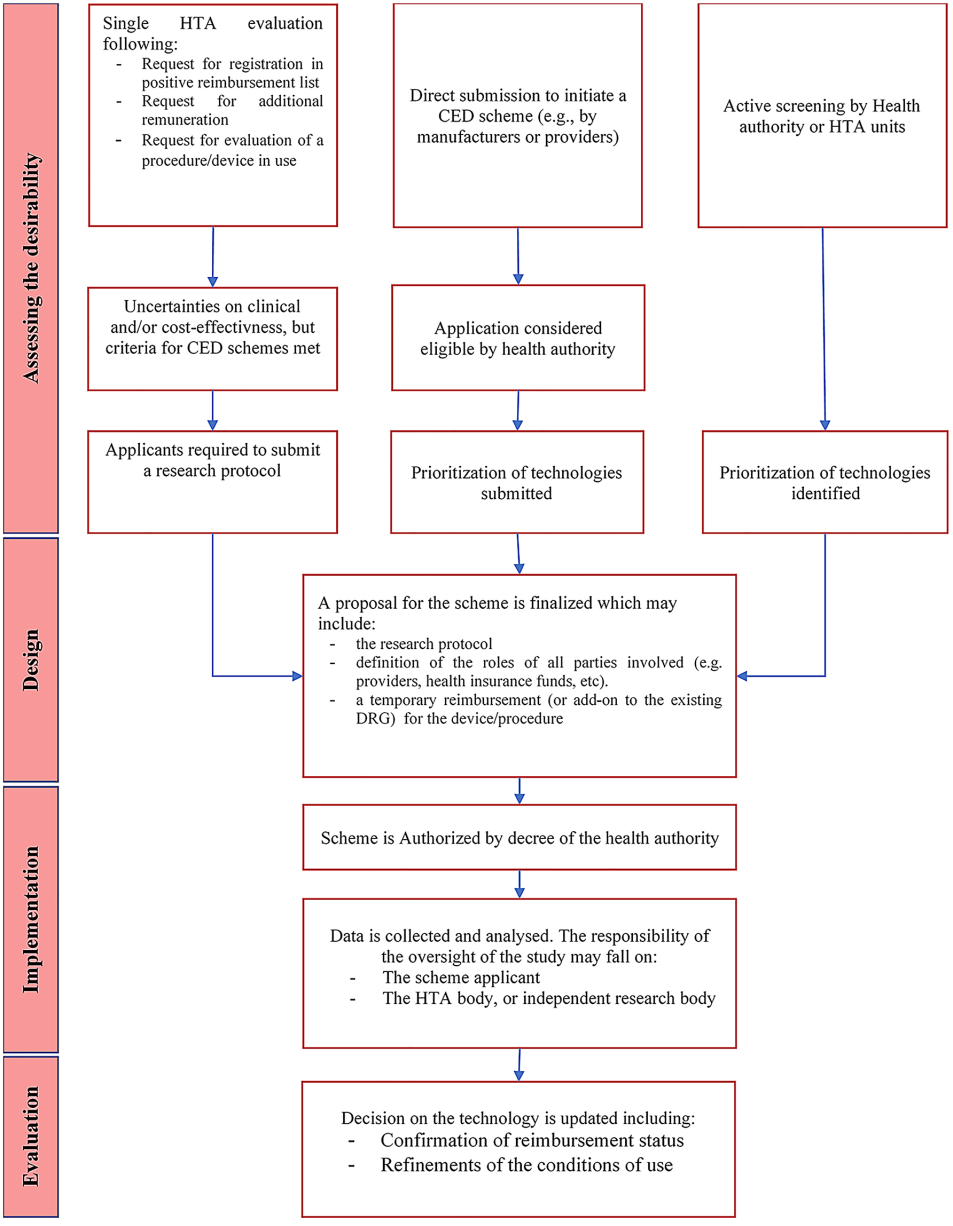
Overview of the main characteristics of CED programmes in Europe

Overall, we identified 78 CED schemes for devices which were ongoing in the last 5 years in Europe. A full overview of the characteristics of these schemes is included in Online Resource 4. Table 3 and Fig. 1 present an overview of how the existing national CED programmes underpinning the individual schemes address the different phases of CED schemes. Our main findings are highlighted below.

**Table 3:** Overview of the characteristics of CED programmes for medical devices in Europe

	England	France	Germany	Netherlands	Spain	Switzerland	Belgium
Name of the CED Policy	Commissioning through Evaluation	Forfeit Innovation (FI)/Post-Registration Studies (PRS) (études post-inscription sur les technologies de santé)	Evaluation of medical examination and treatment methods (§ 137e SGB V)	Conditional admission (Voorwaardelijke toelating)^a^	Postlaunch evidence-generation studies (Estudios de Monitorización”)	Services in evaluation (Leistungen in Evaluation)	Limited clinical application (Beperkte klinische toepassing)
Desirability of schemes							
Technology selection	Proposals for new schemes are co-ordinated by NHS England’s CRGs during a ‘Topic Selection’ phase and assessed by the Clinical Panel that determines which schemes go forward for implementation	FI package: proposals are submitted by manufacturers alone or in partnership with physician’s associations PRS: if, during the assessment of a request for inscription in the LPPR, the CNEDiMTS identifies remaining uncertainties on the technology’s short or long-term outcomes, it can require collection of new data through a PRS	During the evaluation procedure of a diagnostic and therapeutic method, if the opinion of the IQWIG reports that the benefit has not been confirmed, but the method offers the potential of being a treatment alternative. Requests for the evaluation of methods may be put forward by 1) stakeholders organizations for inpatient (§ 137c, SGB V) and outpatient (§ 135 SGB V) care, 2) directly by manufacturers (§ 137e SGB V) or 3) by hospitals, submitting a first request for NUB payment to the InEK (§ 137 h SGB V)	Technologies can be identified in 2 ways: 1) a bottom-up process where parties can submit their own application once a year; and 2) a top down process, where the ZIN recommends, in any negative view following an assessment, whether an intervention can be eligible for conditional admission	Technologies are identified by the National Commission of Provision, Insurance and Financing (CPAF) of the Ministry of Health and selected by the Directorate General of the common portfolio of services of the National Health System (NHS) and Pharmacy (DGPSPh). Topics are usually identified from previous HTA reports from the Spanish Network for Health Technology Assessment and Services of the NHS (RedETS)	Technologies can be identified in 2 ways: 1) following a request for verification that a medical service is effective, appropriate and efficient (WZW criteria), if during the assessment the EAMGK/CFAMA issues a “Yes in evaluation” recommendation; 2) following a direct request from manufacturers or providers for medical devices that need to be listed under the medical device aid list	CED schemes can be initiated top-down following a technology appraisal by the CTIIMH of the RIZIV. Bottom-up requests for the initiation of CED schemes can be submitted by scientist or participating hospitals; however, these schemes can formally only be initiated by the CTIIMH of the RIZIV
Criteria for eligibility and prioritization	The following eligibility criteria must be met: 1) Technology falls within NHS England’s direct commissioning responsibilities 2) The treatment or care pathway shows significant promise; 3) a clinical commissioning policy is published confirming that the technology is not routinely commissioned, or there are significant remaining questions of clinical or cost-effectiveness, and/or outcomes in the routine clinical setting. 4) existing uncertainties will not be answered by current or planned clinical trials. 5) Meaningful new outcome data can be gathered within the likely timescale of a scheme (1–2 years)	FI: requests are accepted if the device is expected to be innovative (4 criteria: 1) the novelty of the device, 2) early dissemination phase, 3) acceptable risk for patients, and 4) promise of significant health improvements or reduction in healthcare costs; and the protocol is considered adequate to answer the identified research questions (article 165.1.1 of the French social security code) PRS: A request for a PRS is done whenever the CNEDiMTS outlines relevant remaining uncertainties (no prioritization)	The new method must have positive promise of benefit, as defined in the German code of procedures: 1) potential replacement of more complex methods; 2) fewer expected side effects, 3) higher expected clinical benefits	10 primary criteria for admissibility to a scheme (yes/no answers, all to be satisfied), and 5 secondary criteria for prioritization (score from 1 to 10). Prioritization criteria include: 1) Disease burden, 2) existence of clinical alternatives, 3) the expected added value of the intervention (health benefits/ economic/organizational/social/ethical impact) 4) existence of other (similar) studies ongoing or planned and 5) The level of evidence of the proposed study (RCTs, observational design, comparative or not-comparative studies)	A quantitative prioritization tool is used. Criteria are defined across 4 domains: 1) Population/end users (e.g., disease burden, frequency of use); 2) Technology (innovativeness, different expectations of use); 3) Safety/adverse effects (e.g., safety issues, undetected adverse effects); 4) organization/costs and other implications (e.g., learning curve, financial impact, organizational or structural impact)	An explicit checklist is used for technology selection and prioritization. Main criteria are: 1) existence of a relevant evidence gap regarding efficiency, safety, cost-effectiveness and conditions of use; 2) interest for the technology (e.g., disease burden, existence of treatment alternatives, significant economic impact); 3) existence of ongoing studies 4) the research proposal can answer the evidence gaps 5) feasibility of a CED scheme for the technology 6) expected positive cost–benefit ratio; and 7) capacity of the new findings to affect coverage decisions	Main criteria used are: 1) the innovativeness of the technology; 2) feasibility of answering the identified research questions within the timeframe of the study
Research design							
Type of CED scheme^b^	Only in research	FI: Only in research, PRS: Only with research	Only with research for Inpatient care, Only in research for outpatient care	Only in research^c^	Only with research (in selected healthcare centres identified at the regional level)	Only with research	Only in research
Types of study design	Mainly prospective observational studies using data collected from existing clinical databases, or by setting up a new registry	FI: Highest level of evidence preferred (e.g., RCTs) PRS: Mainly single-arm, registry based, observational studies	Highest level of evidence preferred (e.g., RCTs)	Highest level of evidence preferred (e.g., RCTs). Furthermore, a supplementary (observational) study may be initiated after the enrolment of the preferred study is completed	Prospective, single-arm observational studies; focus on designs which minimize data collection effort	Preferably RCTs, other designs may be also considered (before-and-after comparisons, case series or comparisons with historical controls)	Prospective observational studies, based on registry data
Implementation							
Funding of the research	NHS England provides funds for service provision to the participating centres and NICE to oversee the scheme. NICE directly commissions an External Assessment Centre for data collection and data analysis	FI: a forfeit payment for the procedure and the associated hospital costs is established at the start of the scheme. Costs of data collection and analysis fall on the scheme applicant PRS: Following the CNEDiMTS appraisal, the device is temporarily listed in LPPR and covered by the social health insurance. Costs of data collection and analysis fall on the manufacturers	G-BA coordinates all phases of the design and implementation of the scheme. The diagnostic and therapeutic method under evaluation is covered by the health insurance. Overheads of the study can be financed by the manufacturer of the device being evaluated or are financed by statutory health insurance via G-BA	The care provided is covered by the basic insurance package. The reimbursement rate is negotiated between the health insurance companies and the participating hospitals and included in a covenant agreement signed by all parties involved in the scheme. The costs of data collection and analysis are covered by the scheme applicants. However, there is the possibility to apply for a research grant at ZonMw	Regional HTA agencies receive funding for data collection, analysis and reporting from the central Ministry of Health. The price of the device is negotiated individually by the regions participating in the scheme. Participating hospitals do not receive extra funding for data collection	The reimbursement of the procedure is covered by the health insurance. Costs of data collection and analysis falls on the manufacturer	Following the recommendation of the CTIIMH to initiate a scheme, the minister of health takes a decision regarding the temporary reimbursement of the care service and the reimbursement methods to be applied. Participating hospitals do not receive any funding for data collection and analysis
Definition of study protocol	The study protocol is developed by the External Assessment Centre in partnership with NICE	FI: The study protocol is directly submitted by the scheme applicant and evaluated by the HAS PRS: The responsibility of defining the protocol falls on the scheme applicants. Authorities only provide broad indications on the type of uncertainties that must be addressed by the scheme, and approve the final version of the study protocol	The key aspects of the study are defined in the directive approving the scheme. The protocol is then refined by the research institution that conducts the study	Development of the study protocol is a direct responsibility of the scheme applicant. ZIN assesses the study proposal in collaboration with the Scientific Advisory Council (WAR) and ZonMw	The study protocol is defined by the regional HTA agencies participating in the data collection	The design of the protocol is a responsibility of the scheme applicant. The proposal is then evaluated and approved by the FOPH	The relevant questions to be answered in the scheme and the set-up of the registry are proposed by the CTIIMH and discussed with the stakeholders involved, to obtain an agreement. Outcomes to be considered are discussed between the expert scientific community and the CTIIMH which also approves the final version of the protocol
Data collection, monitoring. and analysis	The data collection is overseen by the appointed steering group, and supported by the External Assessment Centre. Periodic checks and follow ups are done on the quality and validity of data submitted to ensure meaningful data is being collected. Analysis of the data is done by the external assessment centre and reviewed by NICE	FI and PRS: The responsibility for both the data collection and analysis falls on the manufacturer only. For PRS studies, the CNeDMTs evaluate the quality of the new evidence provided at the time of the planned re-appraisal of the technology	Data collection and analysis are done by an external research institution which has been appointed by G-BA through a public tender, if the overheads are financed via G-BA	The scheme applicant has the main responsibility for data collection and monitoring. The ZIN monitors the course of the scheme and reports it annually to the Minister of Health. ZIN assesses the new evidence provided at the time of the planned assessment of the technology	Data collection and analysis is coordinated by the Regional HTA agencies participating in the scheme. Tasks include checking data completeness and quality, safety surveillance, and yearly reporting to the CPAF on the progress of the scheme	The applicant (provider and/or manufacturer) are the sole responsible for data collection and analysis. Yearly reports have to be reported to the FOPH, showing how the study is proceeding. These reports may inform changes to the scheme or even cause early termination, if issues arise with data collection (e.g., poor quality of the data, slow recruitment)	Data collection is a responsibility of the hospitals that have signed the agreement to participate in the scheme. Depending on the agreement, the hospitals or an external peer-review group/scientific association are responsible for the scientific report
Evaluation							
Existence of ex-ante decision rules for the scheme linking the results of the scheme to a decision on price, reimbursement or use	No	No (both FI and PRS)	No	Agreements regarding the uptake of the intervention, in case of a positive coverage decision at the end of the scheme, or exit strategies in case of a negative opinion (e.g., because the intervention is not effective, or the data quality is considered insufficient to take a decision) are defined in the convenant agreement prior to the start of the scheme	No	No	No
Types of decisions informed by the scheme	Results of the scheme are used for the development of Clinical Commissioning policy for NHS England’s directly commissioned specialised services	FI: Conditional reimbursement is provided only for the duration of the scheme, then devices enter usual evaluation pathways (e.g., a new request by the manufacturer for inscription in the LPPR) PRS: confirmation of the device in the LPPR and refinements of the conditions of use. Financial penalties on the price of the device may be applied in case of poor data quality at reassessment	Confirmation of the reimbursement status	Confirmation of the reimbursement status	Confirmation of reimbursement status under the same conditions of use, changes to the conditions of use or withdrawal of the technology from the benefit package	Confirmation of the reimbursement status, refinements of conditions of use, and changes in the maximum reimbursement rate for the technology or procedure	Confirmation of the reimbursement status

^a^Starting from 2019, conditional admission schemes have started to be gradually replaced by schemes within the new Promising Care Subsidy Fund (PCSF). The main difference between the two programmes concerns the way care provision is reimbursed during a scheme, i.e., directly subsidized in the PCSF rather than covered by the statutory health insurance as in conditional admission schemes. The schemes already ongoing will be completed according to the VT programme described in the Table

^b “^Only in research” are defined as schemes in which a device or procedure is reimbursed only for patients who enrol in a clinical study, whereas “Only with research” schemes are defined as schemes in which a device or procedure is reimbursed for all indicated patients while data are collected in a subset of patients

^c^Conditionally approved care is only covered if the patient participates in the main study. However, patients who are not able to participate can claim the conditionally approved care if they participate in a supplementary ancillary study. CNEDiMTS, French Medical Device and Health Technology Evaluation Committee; CRGs, Clinical Reference Groups (England); CTIIMH, Belgium Implant and Invasive Medical Device Reimbursement Committee;; FOPH, Swiss Federal Office of Public Health; G-BA, German Federal Joint Committee; InEK, German Institute for the Hospital Remuneration System; IQWiG, German Institute for the Hospital Remuneration System; LPPR, List of Products and Services qualifying for Reimbursement (France); RCTs, Randomized Controlled Trials; RIZIV, Belgian Medicines Verification Organisation; ZIN, Netherlands National Health Care Institute; ZonMW, Netherlands organisation for Health Research and Development, Implant and Invasive Medical Device Reimbursement Committee

### Assessing the desirability of a CED scheme

We identified three main ways in which devices are selected for a scheme (Table 3). Firstly, a device can be selected as the direct result of a formal health technology assessment (HTA), if the decision body making the assessment identifies remaining uncertainties on the device (cost-)effectiveness and therefore propose initiation of a scheme. Such HTAs can be conducted for example, in the context of i) a request from a manufacturer to include the device on a positive reimbursement list (e.g., Belgium, France—PRS, the Netherlands and Switzerland); ii) a request from a provider for an extra remuneration of the procedure involving the device, for example on top of an existing diagnosis-related group -DRG tariff (e.g., in Germany); or iii) a request for an evaluation of a procedure or device already in use in clinical practice (e.g., in Belgium, Germany, Spain and Switzerland). Secondly, a device could be selected following an active screening of potential candidates for CED schemes conducted by the decision body or by a committee specifically appointed for this task (e.g., in England or Spain). Finally, a device could be selected following a direct application to initiate a CED scheme by manufacturers or other stakeholders, such as care providers and health insurers with an interest in the device (e.g., in Belgium, France—FI studies, the Netherlands and Switzerland).

In all jurisdictions criteria are used to select and/or prioritize devices for inclusion in a scheme, and decisions are made either through a deliberative process or using an explicit scoring system or checklist. However, a formal assessment of the pros and cons of initiating a scheme, as opposed to other policy decisions, such as providing unconditional coverage, or refusing to adopt the device until better evidence becomes available, was never clearly defined.

### Designing a CED scheme

We identified differences in the design of schemes between countries. For example, Spain and Switzerland mainly operated schemes in which a device is reimbursed for all indicated patients while data are collected in a subset of patients (i.e., only *with* research—OWR), whereas England, the Netherlands and Belgium mainly operated schemes in which a device is reimbursed only for patients who enrol in a clinical study (i.e., only *in* research—OIR). In France schemes were either OIR in the FI programme or OWR in the PRS, whereas in Germany the type of schemes depended on whether the technology was intended for inpatient use (OWR) or outpatient use (OIR).

It is worth noting, however, that within countries, the designs were relatively similar between schemes and appeared not to be tailored to (the specific characteristics of) the device under evaluation or to key sources of uncertainty. Moreover, the study designs were similar between schemes in each country and mainly concerned either observational designs utilizing real-world data, or experimental designs to ensure a high level of evidence (e.g., randomized controlled trials).

### Implementing a CED scheme

We observed differences in the governance of CED schemes as well as in the roles and responsibilities of the stakeholders involved, regarding the development of the research protocol and the subsequent monitoring of the scheme. Overall, we identified two main approaches. In the first approach (e.g., in France, the Netherlands and Switzerland), the responsibility for the development of the study protocol, the monitoring of the scheme and the quality of the generated data relies entirely on the scheme applicants (e.g., the manufacturer or care providers). However, in defining the study protocol the applicants typically must follow the recommendations of the relevant decision body. Usually, the protocol requires formal approval before study initiation, to make sure that it is suitable for addressing the identified uncertainties regarding the device. In the second approach, the responsibility for the development of the study protocol and the quality of the generated data are coordinated centrally (e.g., by HTA agencies), and managed either directly or through third-party research centres (e.g., in Belgium, England, Germany and Spain).

Patient representatives may be involved in the initial assessment phase on the desirability of the scheme (e.g., in Spain or France) or later during the recruitment phase of the study (e.g., in England), but their involvement in the design phase and the development of the protocol was generally limited.

During the scheme, the costs of care provision (including utilization of the device) are usually funded through the public health care system. Specific funding arrangements may be defined at the onset to cover the additional costs of the device or procedure, by either establishing a forfeit or negotiating an add-on to an already existing DRG tariff. However, different arrangements exist for covering the additional costs associated with the research, including the costs of developing the study protocol, scientific monitoring, data collection and analysis. These costs may be either entirely financed with public funds (e.g., in Belgium, England and Spain) or they may be partially or entirely covered by the scheme applicant (e.g., in France, Germany, the Netherlands and Switzerland). Notably, in some cases, funding arrangements also include resources for data collection. In other cases, health care providers are required to perform this task without any additional funding, for example, as a condition of participating in the scheme and gaining market access for the device (e.g., in Spain or Belgium).

### Evaluating a CED scheme

Decisions at the end of schemes mainly concerned the confirmation of the reimbursement status of the device, the refinement of clinical indications or conditions of use. For most of the identified schemes, no ex-ante decision rules that explicitly linked the scheme results to future decisions were defined. In most countries the schemes solely concerned the collection of additional evidence to reduce the identified uncertainties, while the final decision on the reimbursement, coverage or use of the device was integrated in the routine decision-making framework. A notable exception was the Netherlands, where the level of effectiveness that must be demonstrated during the scheme to obtain unconditional reimbursement was predefined at the onset of the scheme, in a covenant agreement signed by all stakeholders. Moreover, the covenant also addressed how to manage the withdrawal of a device in case it proved to be insufficiently effective or the data did not allow an informed decision (e.g., due to poor data quality or inconclusive results).

Notably, all participants reported having no, or only very little experience, with schemes that led to a negative coverage decision. Indeed, of the 24 CED schemes for which information on final decisions were available, coverage was confirmed (or conditional coverage prolonged due to data quality issues) in 22 cases.

### Challenges associated with CED schemes for medical devices

Of the 25 participants, 18 scored the 13 challenges on the six-point Likert scales. Of these, nine were from jurisdictions with CED programmes involving devices, and nine were from jurisdictions with CED programmes involving drugs only. The seven participants who did not score the challenges were from countries without CED programmes.

For most of the assessed challenges, scores were observed across the full range of the Likert scales, indicating no clear patterns in the decision-makers’ perceptions. Table 4 presents the mean and median scores for each challenge. Overall respondents from jurisdictions with CED programme for medical devices tended to give lower scores to most of the challenges as opposed to respondents from jurisdictions without such programmes. However, the low sample size and the variability in responses within each challenge hampered any firm conclusion.

**Table 4:** Assessment of challenges by participants^a^

	Challenge	Participants from countries with CED programmes for medical devices (Belgium, England^b^, France^b^, Germany, Netherlands, Spain, Switzerland)			Participants from countries without CED programmes for medical devices (Bulgaria, Hungary, Ireland, Italy^b^, Poland, Portugal, Scotland, Slovakia)		
		*n*	Mean (SD)	Median (IQR)	*n*	Mean (SD)	Median (IQR)
1	Deciding which medical devices are candidates for CED schemes	9	2.5 (1.17)	2 (2.25)	9	3.78 (1.48)	4 (2.5)
2	Obtaining stakeholder agreement on the scheme	9	2.17 (1.46)	2 (2.75)	8	2.75 (1.83)	2.5 (3.5)
3	Securing funding for the scheme	9	0.89 (1.05)	1 (1.50)	8	3 (1.69)	3 (3.5)
4	Determining the appropriate study design for data collection	9	2.39 (1.45)	2 (2.75)	9	3.33 (1.32)	4 (2)
5	Determining the relevant outcome measure(s) on which data are collected	9	2.61 (1.27)	2 (2.50)	9	2.78 (1.72)	2 (3.5)
6	Dealing with data collection and monitoring	8	2.13 (1.64)	2.5 (3.5)	9	3.78 (1.2)	4 (2.5)
7	Dealing with data analysis	9	1.61 (1.22)	1.5 (2.5)	8	3 (1.51)	3.5 (2.75)
8	Ex-ante definition of decision rule, based on possible outcomes of the scheme	3	3 (1)	3 (2)	8	3.75 (1.58)	4.5 (2.75)
9	Reaching an agreement on price, reimbursement or use of the device at the end of the scheme	5	2.1 (2.13)	2 (4.25)	7	3.57 (1.27)	4 (3)
10	Withdrawing a device from the market when evidence indicates the device is not (cost-) effective	6	3 (0.89)	3 (2)	8	4.5 (1.07)	5 (0.75)
11	Obtaining agreements about the duration of the scheme and the stopping rule	9	1.94 (1.13)	2 (1.25)	8	1.75 (1.49)	1.5 (2.75)
12	Adapting the scheme to account for product modifications or a learning curve	8	1.44 (1.45)	1.5 (2.38)	8	3.25 (1.49)	3.5 (2.75)
13	Dealing with the market entry of similar devices	9	1.83 (1.73)	1 (2.75)	8	2.25 (1.67)	2 (3)

^a^Assessed on a six-point Likert scale (ranging from 0 “not a challenge” to 5 “a major challenge”)

^b^Two participants scored the challenges for this country

Table 5 presents the main factors that, according to the participants, positively or negatively influenced the challenges. Many of the factors identified are common to all technologies and consistent with the existing literature on CED schemes. However, some elements specific to devices could be identified.

**Table 5:** Factors with positive and negative influence on challenges with CED schemes for devices

	Challenge	Factors with positive influence	Factors with negative influence
1	Deciding which medical devices are candidates for CED schemes	There is a structured process leading to the identification of potential candidates for CED schemes Prioritization and inclusion of technologies into a scheme is made according to explicit and shared criteria The suitability of the proposed study protocol is a pre-condition to inclusion of a technology in a scheme The request to provide additional data is applied to all technologies for which relevant evidence gaps are identified during an assessment and the main responsibility of data collection falls on the manufacturers/applicants	HTA processes for devices are less formalized, commissioning mainly occurs at the local level A high number of devices and lack of horizon scanning processes to inform candidates for CED schemes of medical devices Optimal allocation of the funds for CED schemes is hampered by the fact that proposals are evaluated at different times over the year It is not easy to establish whether the available evidence is sufficient to initiate CED scheme or whether it is too early for reimbursement
2	Obtaining stakeholder agreement on the scheme	There exists a well-defined and structured processes for stakeholder engagement All details of the scheme, including the roles and obligations of the stakeholders involved are defined in a contractual agreement before scheme initiation Relationships with clinicians and manufacturers are facilitated if CED schemes are perceived as the only means to use the technology The responsibility to collect the data (and coordinate with participating centres and other stakeholders) fall on manufacturers/applicants	The complexity of CED schemes and the different expectations of the stakeholders involved require a strong and time-consuming coordinating effort For devices, it is more difficult to find patients to participate in public consultations during the scheme (e.g., compared to pharmaceuticals) In countries with small markets manufacturers may have a high bargaining power when discussing the conditions for the schemes
3	Securing funding for the scheme	Fixed budgets for CED schemes are granted on a periodic basis The additional costs of running a scheme fall upon the manufacturers/applicants	Lack of ad hoc funds and/or human resources to run the schemes
4	Determining the appropriate study design for data collection	The health authority can explicitly or implicitly mandate the type of study to be conducted Study design is defined by a third-party research institution CED schemes are mostly relying on routinely collected data A registry on the disease/device is already in place and suitable to answer the research questions	Setting up the research governance is usually complex, with several organizations involved and many practical questions to answer There may be disagreement on study design between the government, the manufacturers and the providers Selecting the centres that will collect data for the schemes may be problematic and time consuming Original patients’ informed consent for registries may not allow subsequent analyses of data
5	Determining the relevant outcome measure(s) on which data are collected	The health authority defines the primary and secondary outcomes. Those responsible for carrying out the research must justify if they do not follow the indication Clinicians and experts are involved from the onset in the definition of the outcomes Previous evidence from the literature or international collaborations (e.g., EunetHTA reports) already outlined the most relevant outcomes	Relevant safety and effectiveness issues are more difficult to identify for devices compared to drugs at the time of the evaluation Patient Reported Outcomes data are generally difficult to collect A balance is required between what outcomes would be desirable and what can be pragmatically collected by the participating centres Different stakeholders may disagree on the relative importance of the outcomes to be collected (e.g. surrogate versus patient relevant outcomes)
6	Dealing with data collection and monitoring	Data collection is based on routinely collected data from electronic sources (e.g., electronic health records) Feasibility of the data collection burden is discussed and agreed among all actors involved at the beginning of the scheme There is interoperability of data across data sources and research centres/providers Continuous follow-up is done to check the quality and validity of data submitted and to ensure meaningful data is being collected	There is less availability of routinely collected outcomes data for devices compared to pharmaceuticals Uncertainties on devices may require the collection of long-term outcome data, incompatible with the length of the scheme Having to deal with many low-volume centres with different experience may affect data quality, and increase the collection effort Hospitals/participating centres may lack incentives to provide timely and high quality data if they do not receive specific funding for this task Recruitment may be slower than expected affecting the time when the scheme reports its results
7	Dealing with data analysis	An independent research body is appointed for data analysis, including quality and risk of bias assessment There is an established experience with data analysis	Difficult to find adequate controls with observational studies Getting the analysis done and timely delivered may be difficult if no additional funds are provided for this task
8	Ex-ante definition of decision rule, based on possible outcomes of the scheme	Schemes are only about collection of new data Decision rules, including stopping rules during the schemes, and management of specific cases at the end of the data collection (e.g., insufficient quality of data, technology not effective) are defined in a contract agreed among all parties involved	Fixed decision rules at the onset may be affected by unforeseen changes in the devices or market dynamics
9	Reaching an agreement on price, reimbursement or use of the device at the end of the scheme	At the end of the scheme, technologies are re-evaluated according to business as usual evaluation procedures	The scheme may not have collected the planned data by the time of the reassessment, or data may be un-conclusory Relevant differences in (cost) effectiveness less clear among similar devices compared to pharmaceuticals
10	Withdrawing a device from the market when evidence indicates the device is not (cost-) effective	An exit strategy in case the technology is not (cost) effective is defined at the onset in a contract agreed between all stakeholders involved Having a well-designed scheme which produces scientifically robust results	Patients and manufacturers may challenge the withdrawal decision and take actions against it The management of explants for implantable devices in case the study outlines safety issues is complex
11	Obtaining agreements about the duration of the scheme and the stopping rule	The duration of the scheme is agreed based on the time that is needed to collect the required data and the characteristics of the disease/technology Continuation of the scheme is linked to periodic monitoring on its progresses	Adopting the stopping rules defined at the onset of the scheme may be difficult when the scheme is ongoing There is a tension between the short life-cycle of devices and the need for long-term outcomes Different perspectives among involved stakeholders (e.g. clinicians, manufacturers, NHS and HTA bodies) Slow recruiting may impact on the time when the study reports its results
12	Adapting the scheme to account for product modifications or a learning curve	The time frame of the scheme is relatively short to avoid product modifications Considerations on the eligibility of a device to a scheme also consider if newer generations of the same devices are expected in the short-term The company shares in advance available information on potential evolutions of the device and these are considered when discussing the study protocol Data on the effect of the learning curve is publicly available	There is little policy experience with how to deal with product modifications and/or learning curves Interpretation of results are confounded by product modifications that occur during the time-frame of the study Existence of a learning curve may complicate the selection process of participating centres in the scheme
13	Dealing with the market entry of similar devices	Schemes evaluate the class of devices or the procedure, not individual devices A scheme can involve multiple devices from different manufacturers Schemes are not comparative in nature. Any similar product entering the market may be requested to provide additional data or not based on their level of evidence Manufacturers of similar devices entering the market after scheme is initiated may be required to provide data to the same nationally-wide registry	Identifying similar devices entering the market is hampered by the difficulty to do horizon scanning for devices More rapid changes in clinical practice with devices compared to pharmaceuticals Inclusion of a new device entering the market when the scheme is ongoing may be more difficult than including it from the beginning

*CED* coverage with evidence developmen

Devices were generally considered to be more difficult to identify and monitor than pharmaceuticals, given that their routes to market are often less clear and may not be observed by those who are responsible for selecting potential candidates for CED schemes. The intrinsic characteristics of devices were also reported to pose additional challenges in the design and implementation of schemes. For example, device-user interactions and the context-specific factors which may affect device performance in the real-world were considered as challenges for the identification of all relevant uncertainty at the time of scheme initiation, and for the definition of the study protocol. In addition, devices may be associated with uncertainties that cannot be easily resolved within a feasible time frame for a scheme, such as uncertainties over the devices’ durability or their long-term performance in patients with different clinical conditions and physiologies. This in turn may increase the tension between the need to pragmatically rely on surrogate endpoints, which are rarely validated for MD procedures, and the relevance of the data collected to inform decision-making at the end of the scheme. In addition, routinely collected data, such as administrative datasets or electronic health records were expected to be less often available, or relevant, for devices, as compared with pharmaceuticals.

Relating to the possibility of product modifications during the timeframe of the scheme, one of the main concerns related to the fact that such modifications could bias the results of the study or compromise the relevance of the new evidence collected. In this respect, being able to anticipate product modifications by means of dialogues with manufacturers and sharing of information was considered a potentially mitigating factor. However, the possibility of product modifications was not perceived by most of the respondents as a major challenge, or something which is likely to occur during the duration of a scheme.

Similarly, about half of the respondents did not consider the possibility that similar products would enter the market during the period of the scheme to be an important challenge. Possible reasons related to the fact that most of the schemes evaluate a class of devices or a procedure rather than a single branded device, or that, even if focussed on a single product, they collected mainly non-comparative data. However, other respondents emphasised the difficulty of anticipating which products would enter the market during the schemes and the possibility that relative effectiveness estimates may not be meaningful anymore by the end of the scheme, as clinical practice changes more rapidly in the context of devices compared to pharmaceuticals.

Finally, with respect to the existence of a learning curve, interviewees acknowledged it as a challenge which affects both the collection and analysis of data, as well as the design of the study, such as deciding on the number of clinical centres authorized to use the device as part of the scheme. However, direct experience with this aspect was generally limited across all respondents.

## Discussion

CED schemes and their application to medical devices are important items on the policy and research agendas. The objectives of this research were to explore the characteristics and use of CED schemes for devices in Europe, as well as the challenges that decision-makers face when designing and operating these schemes. Our study importantly adds to the existing knowledge base by providing a comprehensive and multi-country overview, which was directly informed by surveys with European decision/makers.

We found that 78 device-related CED schemes have been operated over the last 5 years in European countries. However, only seven countries had CED programmes in place for medical devices. To a large extent, this result may reflect the uneven application of HTA within Europe, since it may be difficult to develop a policy for CED schemes without having an established HTA capacity. For example, deciding that more data are required post-launch implies that some form of assessment of clinical or cost-effectiveness has been made. Nevertheless, HTA capacity cannot fully explain these differences, since CED schemes seem to be less frequently used for devices than for drugs [15].

The characteristics of the identified CED programmes underpinning the individual schemes for devices varied between countries, which may reflect local differences in how HTA is organised and practised. For example, schemes were either initiated by the authorities (i.e., Ministry of Health), often as a consequence of the findings of an HTA for the technology, or as a response to a request from a manufacturer. We found similar patterns in the relative responsibilities for the funding of schemes and the design of study protocols although the authorities always played some role in study design, either by outlining a general specification or recommending that an independent research centre be involved. These differences in roles were also found in the aspects of the implementation of schemes, including the collection and analysis of data, which was sometimes the responsibility of the manufacturer and sometimes an independent party.

One aspect that deserves attention is how devices are selected for a scheme. Indeed, CED is not a costless activity and its (opportunity) costs and benefits should be considered alongside other policy options, such as adopting or refusing adoption of the technology, based on currently available data, or negotiating a lower price. Aspects to be considered should include: 1) the expected value of research option(s) in terms of reduced uncertainty; 2) the direct costs of collecting evidence; 3) the opportunity costs of any delay in providing access to the technology because of the scheme; and 4) the existence of any irreversibility in the process (e.g. difficulty to subsequently withdrawal the technology, or difficulty to conduct further research after conditional approval) [1, 13]. However, while all the identified programmes used criteria to identify and prioritize technologies for a scheme, a formal assessment of these aspects was generally missing. Related to the previous point. In many jurisdictions, there does not seem to be an option for choosing among different types of CED schemes, such as OWR and OIR schemes. Nonetheless, also depending on characteristics that are specific to, or particularly relevant for devices (e.g., the existence of irreversible upfront investment costs), there may be cases where either one or the other type of CED scheme would be optimal [13, 18]. As reported in the recent report from the ISPOR good practice Task Force, Value of Information (VOI) analysis may be used to support formal assessments on the opportunity to initiate a CED scheme and the type of scheme which maximizes optimal allocation of healthcare and research funds [19].

In addition, one general finding across all countries was that relatively little attention seemed to be paid to the evaluation of schemes, both *in itinere* during data collection and at the time of the reassessment of the technology once the scheme reported its results. This mirrors the findings of other studies of CED and market access schemes more generally [1, 20, 21] and is obviously an area that requires further attention by policy makers and researchers. Indeed, issues with the quality and timely reporting of data have been mentioned as a factor hampering CED schemes (see e.g., Table 5). For example, in France, where manufacturers are solely responsible for the collection of additional data, the lack of the requested evidence from post-registration studies was often reported in the technology re-appraisals.

The policy responses at the end of a CED scheme for devices may be more complicated than, for example, deciding on whether to include a drug on a formulary or to determine prescribing guidelines, since the reimbursement of devices, and the policies to determine their use, are often linked to the use of broader surgical, or other treatment, interventions. Therefore, policies probably involve adjustments to DRG tariffs, or changes to clinical guidelines, and/or hospital practice more generally. Hence, decision rules and policies for discontinuing the use of devices require attention in this context.

Notably, all participants reported to have no or little experience with refusing to confirm reimbursement at the end of the schemes. While this may reflect the degree and type of uncertainties existing at the beginning of the schemes, it may also signal a certain difficulty in reversing the preliminary reimbursement decision once a technology has entered a scheme [17, 22, 23]. This aspect may be even more relevant if no *ex-ante* criteria for evaluating the schemes were defined, as was the case for almost all schemes for devices in Europe.

Based on our observations of variation in the characteristics of schemes, it is difficult to prescribe a single preferred approach to CED of devices in Europe. Each country has specific local differences in HTA practices, although knowledge on how CED schemes have been used elsewhere can be used to develop local guidance. However, ideally a primary driver of the initiation of CED schemes would be the outcome of HTAs for the technologies concerned, since this can help identify the uncertainties in (cost-) effectiveness that (in principle) could be resolved through CED.

The participants’ perceptions of the various challenges in initiating, designing, implementing, and evaluating CED schemes were varied and did not indicate that, in general, some challenges were substantially more important than others. The reasons for this are unclear, although in some cases the participant’s perception of a given challenge reflected local circumstances. For example, funding was not perceived as a major challenge in settings where public funding was made available, but a major challenge in settings where it was not. In addition, the scores obtained for those challenges that were ‘device specific’ did not differ substantially from those for the other, more generic challenges. While this aspect requires further investigation, our general impression was that some of the low scores given for ‘device specific’ challenges are attributable to a lack of direct experience with addressing these issues, given that the use of CED schemes for medical devices in some European countries is generally quite recent. For example, it has been argued that manufacturers may be reluctant to engage in a scheme and generate new evidence if other competitors entering the market with fast-follower products could also benefit from it [24]. So, one option would be to require that each manufacturer generates the same clinical evidence as for devices already on the market, unless there is compelling evidence of ‘equivalence’ for the new device [24, 25]. However, this option risks a waste of (public) resources in conducting clinical studies that are not strictly necessary. Moreover, the consequences of such a strategy in terms of competitiveness, market prices and eventually access of potentially valuable devices to patients remain largely unexplored.

We observed that the scores for the challenges were lower for respondents in countries where there was direct experience in CED for devices, as compared with those having experience with CED for drugs only. However, although the numerical differences in the scores were substantial, the small sample size means that no firm conclusions can be drawn. This could be explored in further research by comparing decision-makers’ perceptions before and after operating CED schemes and relating these perceptions to the general (HTA) infrastructure in a country.

We used a combination of methods to obtain insights in the use of and challenges related to CED schemes in the relatively understudied context of devices, including a large set of European countries. The insights obtained allow learning from experiences across countries and increase the chances of having successful CED schemes in the future, by highlighting how decision makers perceive and deal with specific challenges. Nonetheless, some limitations also need highlighting. First, although we studied experiences in many European countries, we cannot be sure that our overview is complete as some countries were not included in the study. Moreover, although in each country we interviewed the person we considered to be most knowledgeable about CED schemes, we cannot be sure that the views of the participants are representative of the views of decision-makers more generally. Additionally, we focussed on the detailed perceptions of decision-makers, with a focus on HTA agencies at the national or regional level and (some) national payers because recent research suggests that decision-makers may be hesitant to engage in CED schemes [5]. This makes them not only a relevant source for the current study in terms of knowledge, but also in articulating (potential) challenges and difficulties with applying such schemes. Future studies could nonetheless supplement this with information on the perceptions of other stakeholders, such as clinical professionals, patient organisations, local payers/decision makers, and manufacturers. Finally, our focus was on schemes initiated at the national or regional level. In addition, some schemes involving devices may be negotiated at the local level directly between providers and manufacturers. Many of these may be ‘pay for performance’ schemes, but some could be characterized as CED schemes. These schemes were outside the scope of our current study, but their characteristics and performance are nonetheless important to investigate further.

## Conclusions

CED schemes for medical devices offer a promising tool to increase value for money in health care. While they are currently used in Europe, this study has shown experience with these schemes to be limited to a relatively small number of countries. Moreover, considerable variation exists between countries in how schemes are initiated, designed, implemented, and evaluated.

While the identified challenges in using CED schemes were perceived differently, none of them was unanimously considered insignificant. Hence, all challenges should be considered when initiating CED schemes in a given country. Our recommendation is that each jurisdiction embarking on CED schemes for devices should undertake its own ‘risk assessment’*,* using our list of challenges as a starting point, and considering for each of them the factors that decision-makers in this study outlined as having either a positive or negative influence. If a given challenge is considered to be important locally, the highlighted experiences of other countries in this study can help in addressing or overcoming them. That way, this study directly contributes to making CED schemes for devices a more effective policy option in the future.

## Supplementary Information

Below is the link to the electronic supplementary material.

Supplementary file1 (PDF 197 KB)

Supplementary file2 (PDF 84 KB)

Supplementary file3 (PDF 34 KB)

Supplementary file4 (XLSB 84 KB)

## Data Availability

The full database with information on the 78 CED schemes collected is provided in Online Resource 4.
