# Supplementary material for: Coverage with evidence development schemes for medical devices in Europe: characteristics and challenges
Source: Eur J Health Econ. 2021 Jun 12;22(8):1253–73. doi: 10.1007/s10198-021-01334-9 (PMC8526454; doi:10.1007/s10198-021-01334-9)
Supplement: Supplementary file 1 — Supplementary file1 (PDF 197 KB) [file 10198_2021_1334_MOESM1_ESM.pdf]

Electronic Supplementary Material 1 - Structured interview guide

SECTION A

Name of interviewee: ____
Position: ____ ____
Years of relevant work experience: ________________________________
Name of organization: ____

NOTE: only summary data will be reported and individual respondents will not be identified

1. At present in your organization, do you implement CED schemes for health technologies?

- Yes, for medicines only ☐
- Yes, for medical devices only ☐
- Yes, for other technologies ☐ Please specify:__________________________________
- Yes, for all technologies ☐
- No ☐

<follow-up questions 2-6 if answer to Q1 is "Yes" (any technology)>

2. If CED schemes for medical devices are currently implemented, please provide the names of the existing policies or programs underpinning the schemes

3. If CED schemes for medical devices are not currently implemented, has your organization ever considered implementing them?

- No, never considered ☐
- Yes, in the past but it was later abandoned/discontinued ☐
- Yes, in the present/ongoing ☐

4. Please explain your answer

5. Are there any guidelines available on how to design or implement CED schemes that are used in your organization?

- Yes, for all technologies ☐
- Yes, for medical devices only ☐
- Yes, for medicines only ☐
- Yes, for other technologies ☐
- No, guidelines do not exist ☐
- No, but guidelines are currently under development ☐

6. If guidelines exist for medical devices, please provide a reference or link to the guidelines.

<follow-up questions 7-8 if answer to Q1 is 'No'>

7. Has your organization ever considered implementing CED schemes for medical devices?

- No, never considered ☐
- Yes, in the past but it was later abandoned/discontinued ☐
- Yes, in the present/ongoing ☐

8. Please explain your answer.

SECTION B

Based on the literature on CED schemes for medical devices, we have identified challenges that may affect a successful design and implementation of schemes. We now want to ask your thoughts on these challenges, and explore how these are addressed in your organization. When answering questions in this section, please consider CED schemes for medical devices only.

1. Please rate the following challenges on a scale of '0' (Not a challenge) to '5' (Major challenge)

(1) Deciding which medical devices are candidates for a CED scheme	0	1	2	3	4	5
To what extent is this a challenge for your organization? (0 = not a challenge, 5 = major challenge)	☐	☐	☐	☐	☐	☐
Please explain your answer:						
If CED schemes for MDs are currently implemented, what criteria are used to decide on which devices are candidates?						

(2) Getting stakeholder agreement on the scheme (e.g., Ministry of Health, manufacturers, hospitals, clinicians, patient associations)	0	1	2	3	4	5
To what extent is this a challenge for your organization? (0 = not a challenge, 5 = major challenge)	☐	☐	☐	☐	☐	☐
Please explain your answer:						
If CED schemes for MDs are currently implemented, what efforts do you make to engage with stakeholders and get an agreement on the scheme?						

(3) Securing funding for the scheme	0	1	2	3	4	5
To what extent is this a challenge for your organization? (0 = not a challenge, 5 = major challenge)	☐	☐	☐	☐	☐	☐
Please explain your answer:						
If CED schemes for MDs are currently implemented, how is the funding secured to run the scheme?						

(4) Determining the appropriate study design for data collection (e.g., RCT, registry, audit)

	0	1	2	3	4	5
To what extent is this a challenge for your organization? (0 = not a challenge, 5 = major challenge)	☐	☐	☐	☐	☐	☐
Please explain your answer:						
If CED schemes for MDs are currently implemented, how is the study design defined and agreed among all relevant stakeholders?						

(5) Determining the relevant outcome measure(s) to be collected in the scheme	0	1	2	3	4	5
To what extent is this a challenge for your organization? (0 = not a challenge, 5 = major challenge)	☐	☐	☐	☐	☐	☐
Please explain your answer:						
If CED schemes for MDs are currently implemented, how are decisions concerning the outcomes measures made? How is agreement reached among stakeholders?						

(6) Dealing with data collection and monitoring (e.g. who collects the data? Address risk of incomplete/partial data)	0	1	2	3	4	5
To what extent is this a challenge for your organization? (0 = not a challenge, 5 = major challenge)	☐	☐	☐	☐	☐	☐
Please explain your answer:						
If CED schemes for MDs are currently implemented, who is responsible for data collection and quality monitoring?						

(7) Dealing with analysis of the data (e.g. who performs the analysis, how is the risk of bias addressed)	0	1	2	3	4	5
To what extent is this a challenge for your organization? (0 = not a challenge, 5 = major challenge)	☐	☐	☐	☐	☐	☐
Please explain your answer:						

If CED schemes for MDs are currently implemented, who is responsible for the analysis of the data collected?

(8) Determining the decision rule prior to the start of the scheme, based on the outcome of the scheme (e.g. reimbursement status, price change or refund)

	0	1	2	3	4	5
To what extent is this a challenge for your organization? (0 = not a challenge, 5 = major challenge)	☐	☐	☐	☐	☐	☐
Please explain your answer:						
If CED schemes for MDs are currently implemented, by what process is the decision rule determined (or not)? (if a decision rule is established please detail the criteria used)						

(9) Reaching an agreement on price, reimbursement or use of the device at the end of the scheme.

	0	1	2	3	4	5
To what extent is this a challenge for your organization? (0 = not a challenge, 5 = major challenge)	☐	☐	☐	☐	☐	☐
Please explain your answer:						
If CED schemes for MDs are currently implemented, how is an agreement reached on prices, reimbursement or use of a device at the end of a scheme?						

(10) Withdrawing devices from the market when found not to be clinically or cost-effective.

	0	1	2	3	4	5
To what extent is this a challenge for your organization? (0 = not a challenge, 5 = major challenge)	☐	☐	☐	☐	☐	☐
Please explain your answer:						

If CED schemes for MDs are currently implemented, what actions do you take to withdraw devices that are not found to be effective or cost-effective? How do you deal with potential reactions from physicians, patients and the general public?

(11) Agreeing the length of the scheme or stopping rule

	0	1	2	3	4	5
To what extent is this a challenge for your organization? (0 = not a challenge, 5 = major challenge)	☐	☐	☐	☐	☐	☐
Please explain your answer:						
If CED schemes for MDs are currently implemented, how are the duration of the scheme and/or the stopping rule defined and agreed among relevant stakeholders?						

(12) Adapting the scheme to deal with product modifications or the existence of a learning curve

	0	1	2	3	4	5
To what extent is this a challenge for your organization? (0 = not a challenge, 5 = major challenge)	☐	☐	☐	☐	☐	☐
Please explain your answer:						
If CED schemes for MDs are currently implemented, is the possibility of incremental innovations and/or the existence of a learning curve addressed in the schemes? If so, how?						

(13) Dealing with similar products entering the market (e.g., should they be entered into the scheme,

could they undermine the scheme?)	0	1	2	3	4	5
To what extent is this a challenge for your organization? (0 = not a challenge, 5 = major challenge)	☐	☐	☐	☐	☐	☐
Please explain your answer:						
If CED schemes for MDs are currently implemented, how do you deal with similar products entering the market during the scheme?						

2. Are there other factors in your view that may help or hinder the successful implementation of CED schemes?

SECTION C

NOTE: Please include only CED schemes on medical devices that are currently ongoing or were terminated in the past 5 years. If information is not available, please enter "Not Available", if information cannot be made public, please enter "Classified".

Please complete a separate form for each CED scheme for medical devices

1. Basic information on the scheme

1. 1 Year of initiation of scheme: ____

1.2 ID of the scheme: ____

1.3 Description of the product(s) and/or procedure(s) involved:

1.4 Disease/Indication(s) included in the scheme:

1.5 Manufacturer(s) involved:

1.6 Actual status of the scheme (choose one of the answer options):

☐ Scheme agreed, data collection to be initiated
☐ Data collection active
☐ Data collected, re-assessment to be done
☐ Data collected, re-assessment done

1.7 How was this MD selected for the scheme:

1.8 Health care organization/insurer/payer promoting the scheme:

1.9 Agency running the scheme (if not insurer/payer):

1.10 Purpose of scheme (choose one between the suggested options or enter a new one):

☐ To confirm reimbursement status at the end of the scheme (e.g. if effectiveness or cost-effectiveness are confirmed, the technology will receive an unconditional reimbursement)
☐ To inform price decisions (e.g. if agreed clinical outcomes are not reached at the end of the scheme, price discounts are increased by a pre-agreed amount)
☐ To confirm use of the device for specific subpopulations and/or indications
☐ To ensure effective and/or cost-effective use of the device in clinical practice (e.g., payment or reimbursement linked to individual patients' outcomes)
☐ To ensure appropriateness and quality of care (e.g., reimbursement conditional on providers compliance with clinical guidelines, or with pre-defined patients selection criteria)
☐ ____

2. Design of the scheme

2.1 Agreed length of scheme (years): ____

2.2. Description of the agreement (e.g., refund in case of non-response/treatment failure):

2.3 Key sources of uncertainty at scheme initiation (choose among the suggested options or enter new ones):

☐ The efficacy/effectiveness of the technology in a tested population as compared to current standard of care
☐ The relative efficacy/effectiveness in the real target population and/or in different population subgroups
☐ The effects on long-term patient relevant outcomes (versus surrogates outcomes used in clinical studies)
☐ The effect of physicians learning curves on patient outcomes and/or costs
☐ The risk of adverse events and/or adherence problems
☐ Uncertainties related to the true budget impact of introducing the technology
☐ Uncertainties related to the true cost-effectiveness of the technology
☐ Uncertainties related to the organizational impact of the technology
☐ ____
☐ ____
☐ ____

2.4 Design of the study (choose between the suggested options or enter a new one):

☐ Randomized controlled trial
☐ Cohort study

☐ Set up of a registry
☐ ____

2.5 Primary outcomes being measured in the scheme:

1) ____
2) ____
3) ____
4) ____
5) ____

2.6 Secondary outcomes being measured in the scheme:

1) ____
2) ____
3) ____
4) ____
5) ____

2.7 Source of funding for scheme:

3. Outcomes of the scheme

3.1 Public source of evidence about the scheme, if any (e.g. peer-reviewed articles, manufacturer's website, public registries):

3.2 Results (if scheme completed):

a) evidence generated by scheme

b) decision(s) made as a result of the scheme

3.3 Overall impression on successful aspects of the scheme:

3.4 Overall impression on failure aspects of the scheme:

3.5 Is there anything you would do differently when designing/applying a CED scheme for this type of MD in the future?

3.6 Other observations about the scheme:

