# Supplementary material for: Coverage with evidence development schemes for medical devices in Europe: characteristics and challenges
Source: Eur J Health Econ. 2021 Jun 12;22(8):1253–73. doi: 10.1007/s10198-021-01334-9 (PMC8526454; doi:10.1007/s10198-021-01334-9)
Supplement: Supplementary file 2 — Supplementary file2 (PDF 84 KB) [file 10198_2021_1334_MOESM2_ESM.pdf]

Electronic Supplementary Material 2 – List of challenges included in the questionnaire to decision-makers as modified from Reckers-Droog et al.

#	Reckers-Droog et al.	#	Challenges included in the questionnaire
1	Deciding on whether a CED scheme is required	1	Deciding which medical devices are candidates for CED schemes
2	Information asymmetry between payers and manufacturers about the potential real-world performance of a technology		
3	Lengthy and complex negotiations	2	Obtaining stakeholder agreement on the scheme
4	Lack of transparency		
5	Lack of governance		
6	Stakeholder involvement		
7	Obtaining funding	3	Securing funding for the scheme
8	Understanding the relevant uncertainties and risks	4	Determining the appropriate study design for data collection
9	Defining the decision problem		
10	Data requirements		
11	Obtaining informed consent		
12	Ethical issues		
13	Identifying meaningful outcomes	5	Determining the relevant outcome measure(s) on which data are collected
14	Quality of the data	6	Dealing with data collection and monitoring
		7	Dealing with data analysis
		8	Adapting the scheme to account for product modifications or a learning curve
15	Ex-ante definition of a final decision rule	9	Ex-ante definition of decision rule, based on possible outcomes of the scheme
16	Deciding on when a CED is considered successful	10	Reaching an agreement on price, reimbursement or use of the device at the end of the scheme
17	Withdrawing a technology	11	Withdrawing a device from the market when evidence indicates the device is not (cost-) effective
18	Defining an adequate duration for a scheme	12	Obtaining agreements about the duration of the scheme and the stopping rule
19	Market entry of new technologies	13	Dealing with the market entry of similar devices
20	Economies of scale in the management of CED schemes and the difficulties small countries may have in applying CED schemes because of the associated costs and monitoring mechanisms		This challenge was considered transversal to all included challenges and covered in section A of the questionnaire.

In order to reduce the participants' burden when attending the interview, the original list of challenges reported in Reckers-Droog et al.[1] *was reduced to 13 challenges by grouping some of the reported items into broader overarching challenges. For example, the challenges regarding the lengthy and complex negotiations to reach consensus on a scheme, the lack of transparency and lack of governance, as well as the challenges related to involving relevant stakeholders were grouped into the broader item "Obtaining stakeholder agreement on the scheme".*
Nonetheless, the challenge regarding the quality of the data collected in a scheme was expanded to explicitly address three separate issues: dealing with data collection and monitoring, dealing with data analysis, and dealing with data issues when accounting for product modifications or the existence of a learning curve effect.
Challenge #20 in Reckers-Droog et al. was considered as a broader challenge which may constitute a barrier to the implementation of CED programmes for devices in the first place. As such, we did not discuss this as a separate challenge,

[1] Reckers-Droog, Vivian, et al. "Challenges with coverage with evidence development schemes for medical devices: A systematic review." Health Policy and Technology 9.2 (2020): 146-156.

but we covered it in section A of the questionnaire where we asked to decision makers the reasons for not using CED schemes for devices.
