# Supplementary material for: Coverage with evidence development schemes for medical devices in Europe: characteristics and challenges
Source: Eur J Health Econ. 2021 Jun 12;22(8):1253–73. doi: 10.1007/s10198-021-01334-9 (PMC8526454; doi:10.1007/s10198-021-01334-9)
Supplement: Supplementary file 3 — Supplementary file3 (PDF 34 KB) [file 10198_2021_1334_MOESM3_ESM.pdf]

Electronic Supplementary Material 3. Overview of the countries, jurisdictions and institutions of the participants to the survey

Country	Jurisdiction	Type of institution	Institution of the participant
Austria		National decision body	Ludwig Boltzmann Institute for Health Technology Assessment (LBI-HTA)
Belgium		National/regional decision body	National Institute of Health and Disability Insurance (RIZIV)
Bulgaria		University	Medical University Sofia
Czech Republic		University	Czech Technical University in Prague
Denmark		Hospital	Odense University Hospital
England		National decision body	National Institute of Health and Care Excellence (NICE)
Finland		National decision body	Finnish National Institute for Health and Welfare[a].
France		National decision body	French National Authority for Health (HAS);
		National decision body	French Medicine Pricing Committee (CEPS)
Germany		National decision body	The Federal Joint Committee (G-BA)
Greece		Hospital	Onassis Cardiac Surgery Center (OSCS)
Hungary		Health insurance	National Health Insurance Fund of Hungary
Ireland		National decision body	Health Information and Quality Authority (HIQA)
Italy	National level	National/regional decision body	Italian Medicine Agency (AIFA)
Italy	Emilia Romagna Region	Regional decision body	Clinical Governance area - Emilia Romagna Region
The Netherlands		National decision body	National Health Care Institute (ZIN)
Norway		Hospital	Norwegian Hospital Procurement Trust, Division Pharmaceuticals (LIS)

Poland		National decision body	National Health Fund
Portugal		National decision body	National Authority of Medicines and Health Products (Infarmed)
Scotland		National decision body	Health Improvement Scotland
Slovakia		Health insurance	Union Health Insurance
Spain	Basque Region	Regional decision body	Basque Office for Health Technology Assessment (Osteba)
Sweden		University	Linköping University
Switzerland		National decision body	Federal Office of Public Health (FOPH)

[a]Until three months before the interview, actually Helsinki University Hospital (HUS)
